# STING inhibits viral lytic reactivation and cell growth in primary effusion lymphoma

**DOI:** 10.3389/fimmu.2026.1823240

**Published:** 2026-05-05

**Authors:** Tiffany S. Nelson, Cathrine Pacini, Amy Nguyen, Travis M. Zeigler, Alexa Ziff, Kimberly Paulsen, Alayna Simpson, Sumita Bhaduri-McIntosh, Zhe Ma

**Affiliations:** 1 Department of Molecular Genetics and Microbiology, College of Medicine, University of Florida, Gainesville, FL, United States; 2 Division of Infectious Diseases, Department of Pediatrics, College of Medicine, University of Florida, Gainesville, FL, United States; 3 Department of Biochemistry and Molecular Biology, College of Medicine, University of Florida, Gainesville, FL, United States; 4 UF Health Cancer Institute, University of Florida, Gainesville, FL, United States

**Keywords:** agonist, EBV, innate immunity, KSHV, primary effusion lymphoma, STING, viral cancer

## Abstract

Kaposi’s sarcoma-associated herpesvirus (KSHV) is a DNA virus linked to multiple malignancies associated with compromised immunity, such as Kaposi’s sarcoma (KS) and primary effusion lymphoma (PEL). These malignancies are strongly linked to HIV coinfection and remain major oncogenic risks even with advanced antiretroviral therapy. Current treatments are limited and often non-curative, highlighting the need to define host pathways that constrain KSHV replication and tumor progression. STING (Stimulator of Interferon Genes) is an essential mediator of antiviral and antitumor immunity. STING has been shown to restrict KSHV reactivation in an artificial iSLK-based cell model, but further validation in more physiologically relevant models is needed. Moreover, STING’s antitumor role has been widely evaluated in multiple types of cancer in clinical trials, but this remains poorly defined in KSHV-related cancers. In this study, we profiled multiple patient-derived PEL cell lines and found variable STING expression patterns. In comparison to the other PEL cells, BC3 had remarkably low STING expression. When stimulated with diABZI, a STING agonist, BCBL1’s ability to reactivate from latency was robustly repressed by STING activation, reflected by attenuated viral gene transcription, protein expression, genome replication, and virion production. In contrast, the lytic reactivation status in BC3 was only minimally attenuated. Consistently, diABZI treatment restricted cell growth in multiple PEL cell lines, but BC3 showed minimal response. Additional loss- and gain-of-function experiments further supported an association between STING and reduced PEL cell growth, as H151 (STING inhibitor) increased BCBL1 growth whereas STING overexpression suppressed growth in both BCBL1 and BC3 cells. These data support STING pathway competence as an innate immune barrier for KSHV lytic reactivation and tumor progression. To assess the generalizability of STING’s antitumor role, we utilized EBV-transformed lymphoblastoid cell lines (LCLs) with variable cGAS or STING protein levels. Consistently, the responsiveness of LCLs to diABZI aligned with STING pathway competence, with minimal effects on growth and viability when STING expression was low or deficient. Overall, this work links STING pathway competence to both antiviral control of KSHV reactivation and reduced lymphoma growth, suggesting a potential new approach to limit KSHV pathogenesis.

## 1 Introduction

Kaposi’s sarcoma-associated herpesvirus (KSHV) is the etiological agent of Kaposi’s sarcoma, primary effusion lymphoma, and multicentric Castleman’s disease (1–3). These malignancies remain a major clinical challenge, particularly in immunocompromised individuals, including people living with HIV, transplant recipients, or older adults (4–7). Unfortunately, there are currently not clinically approved KSHV-specific vaccines or curative therapies (8, 9), underscoring an urgent need to better define host immune control mechanisms and develop effective treatment strategies.

KSHV establishes persistent infection in the host and poses lifelong oncogenic risk, which is closely linked to its biphasic life cycle with latent and lytic phases (10). Following primary infection, KSHV rapidly circularizes its genome into an episome, establishing a persistent latency within the cell (11, 12). Nevertheless, lytic reactivation has been observed in KSHV-positive tumors and can be triggered by hypoxia, oxidative stress, and histone deacetylase inhibitors, leading to infectious virion production and viral dissemination (13–16). Together, these intertwined processes support two critical aspects of KSHV pathogenesis, viral production and tumor growth. While latent viral oncogene programs promote tumor cell survival and proliferation, lytic replication supports viral transmission and can shape the tumor microenvironment (17–21). Accordingly, approaches that can simultaneously restrict viral replication and reduce tumor cell growth are particularly attractive in virus-driven cancers.

One host defensive mechanism positioned at the intersection of antiviral defense and tumor control is the cytosolic DNA-sensing cGAS-STING pathway (22–25). Upon sensing double-stranded DNA, cyclic GMP-AMP synthase (cGAS) catalyzes the formation of the cyclic dinucleotide 2′3′-cyclic GMP-AMP (2’3’-cGAMP) from ATP and GTP (22, 23). 2’3’-cGAMP binds the stimulator of interferon genes (STING) in the endoplasmic reticulum and triggers its ER-Golgi trafficking (24–26). At the Golgi, STING is palmitoylated and recruits TANK-binding kinase 1 (TBK1), enabling interferon regulatory factor 3 (IRF3) activation and induction of type I interferons and cytokines, which then signal through Janus kinase/signal transducer and activator of transcription (JAK/STAT) to drive interferon-stimulated genes (ISGs) with antiviral effector functions (27–31).

Beyond antiviral defense, STING signaling has also been implicated in antitumor immunity and is a major focus of cancer immunotherapies (32–34). STING-driven type I interferon and ISG induction has been associated with T-cell infiltration and dendritic-cell cross-priming within the tumor microenvironment and contributes to radiation-induced immune responses (35–39). In addition to promoting antitumor immunity, STING activation also drives intrinsic apoptosis, autophagy, and senescence in cancer cells (40–43). Accordingly, STING agonists represent an emerging therapeutic option, and multiple synthetic STING agonists are currently involved in clinical trials for non-viral cancers (44–48). For example, diamidobenzimidazole (diABZI) exhibits robust antiviral impact against a wide variety of viruses, including herpes simplex virus-1, coronaviruses, influenzaviruses, and picornaviruses (49–57). Additionally, the anticancer effects of diABZI have been described in mouse tumor models (58, 59).

The cGAS-STING pathway has been shown to restrict KSHV lytic replication in iSLK.219 or iSLK.BAC16 systems (60–64). Knockdown of cGAS or STING results in elevated viral lytic gene expression and infectious virion production. Additionally, multiple virally encoded proteins or miRNAs have been implicated to repress this pathway to facilitate viral infection, highlighting STING’s antiviral role against KSHV (60, 63–65). However, these discoveries require further validation in more physiological models, such as patient-derived PEL models. Furthermore, STING’s antitumor role remains largely unexplored in KSHV-related cancers. Thus, it is important to explore both antiviral and antitumor aspects of STING signaling in our PEL models and evaluate potential interventions of pharmacologic STING activation using STING agonists.

Out of eight patient-derived PEL cell lines, we found that BC3 expressed markedly reduced STING protein (hereafter referred to as STING^low^), revealing a suppressed state of STING signaling. In the BCBL1-based cell model, pretreatment with diABZI (a STING agonist) prior to reactivation caused global reduction of KSHV lytic gene transcription, KSHV genome replication, and virion production. In contrast, we only observed minimal attenuation of lytic replication in the BC3 cell line. While western blot showed that diABZI can activate STING in BC3 cells, this activation was insufficient to mount robust innate antiviral responses. Beyond antiviral defense, diABZI treatment resulted in decreased cell growth and cell viability in a dose-dependent manner in multiple PEL cell lines, but this effect was removed in STING^low^ BC3. Complementary inhibition and re-expression experiments further supported a tumor cell-intrinsic growth-restraining role for STING in PEL. Accordingly, diABZI induces cleaved caspase-3 in BCBL1 but not BC3, indicating that STING activation promotes apoptosis in PEL. Interestingly, reduced or absent cGAS-STING expressions were also observed in multiple Epstein-Barr virus (EBV)-transformed lymphoblastoid lines. Consistent with the PEL discovery, diABZI similarly impaired cell growth and cell viability in STING-expressing LCLs, but not in STING-deficient LCLs. Collectively, our results suggest that STING serves as an important antiviral and antitumor barrier against KSHV and its related cancers. Furthermore, pharmacological STING activation may represent a host-directed potential strategy for KSHV-associated cancers and other viral cancers.

## 2 Materials and methods

### 2.1 Cell culture

The following KSHV-positive PEL cell lines were maintained in RPMI (Corning, Cat#10-040-CV), 20% heat-inactivated fetal bovine serum (Gibco, Cat#10437-028), and 1% penicillin and streptomycin (Corning, Cat#30-001-CL) at 37 degrees C and 5% CO_2_: BC1, BC2, and BCP1 (all gifted from Dr. Damania Blossom, UNC-Chapel Hill); HBL6, JSC1, and VG1 (all gifted from Dr. Eva Gottwein, Northwestern University). BCBL1, BC3, BJAB, BCBL1-TRex-RTA-Luciferase (gifted from Dr. Dirk Dittmer, UNC-Chapel Hill) (66, 67), and EBV-positive lymphoblastoid cell lines LCL9-23A, LCL9-23B, LCL-1, and LCL-2 were maintained in similar conditions using 10% heat-inactivated fetal bovine serum. LCL9-23A and LCL9-23B were recently generated, while LCL-1 and LCL-2 were previously published (68–70). To minimize potential long-term culture effects, we performed experiments within the first two passages upon thawing the cells.

### 2.2 Human subjects and ethics statement

Peripheral blood mononuclear cells were isolated from the blood of healthy human volunteers and LCLs generated as described previously (68, 69). Blood was drawn after obtaining written consent. The study of human subjects was approved by the institutional review board at the University of Florida.

### 2.3 Reactivation of KSHV in primary effusion lymphoma cells

BCBL1 cells were treated with 2 µM diABZI STING agonist-1 [3HCL] prepared in PBS (MedChemExpress, #HY-112921B) for 24 hours prior to lytic induction. BCBL1-TRex-RTA-luciferase (BCBL1-trex-luc) and BC3 cells were treated with 5 µM diABZI for 24 hours prior to reactivation. BCBL1 and BC3 cells were reactivated with 1mM sodium butyrate (NaB) and 25 ng/mL 12-O-Tetradecanoyl-phorbol-13-acetate (TPA). Doxycycline was added at 1ug/mL to induce lytic reactivation in BCBL1-trex-luc cells. Cells were harvested at 0, 48, or 72 hours post-reactivation.

### 2.4 Real-time quantitative PCR

Total RNA was isolated with the Monarch^®^ Spin RNA Isolation Kit (New England Biolabs #T2110), and cDNA was synthesized with a cDNA synthesis kit (MedChemExpress, #HY-K0510), according to the manufacturer’s protocol. qPCR was performed using the SYBR Green qPCR master mix (MedChemExpress, #HY-K0501). Fold change (relative expression) in gene expression was calculated using the 2^−ΔΔCt^ quantification method and expression levels were normalized to 18S or GAPDH. Primers for human genes: 18S forward: 5’-TTCGAACGTCTGCCCTATCAA-3’, reverse: 5’-GATGTGGTAGCCGTTTCTCAGG-3’; cGAS forward: 5’-TAACCCTGGCTTTGGAATCA-’3, reverse: 5’-TAGCCGCCATGTTTCTTCTT-3’; GAPDH forward: 5’-GTCTCCTCTGACTTCAACAGCG-3’, reverse: 5’-ACCACCCTGTTGCTGTAGCCAA-3’; STING forward: 5’-GAGCAGGCCAAACTCTTCTG-3’, reverse: 5’-TGCCCACAGTAACCTCTTCC-3’. Primers for KSHV genes: K8.1 forward: 5’- GGTTGGAGTGGACAGGTTTATC-3’, reverse: 5’- CACGGTTACTAGCACCACTATTT-3’; ORF39 forward: 5’-GTGGGAGTATTCGTGGGTTATC-3’, reverse: 5’-GGTGAACAGTCGGAGTTCTATC-3’; ORF57 forward: 5’-CTGTGTCCTCCTCTGAGTTTG-3’, reverse: 5’-GCAGGGAGTCTGAGTTTCTTTA-3’.

### 2.5 KSHV whole transcriptome

RNA was extracted from duplicate samples to synthesize cDNA. Eighty-eight KSHV viral transcript levels were analyzed using a real-time qPCR-based KSHV transcriptome array as previously described (60, 71). The RT-qPCR primer set was designed for each KSHV open reading frame. Expression levels were normalized to 18S to yield dCT as a measure of relative expression. Following supervised clustering, a heat map and dendrogram depicted by brackets were generated. Higher transcript expression levels are indicated by red and lower expression levels by blue as shown in the key.

### 2.6 Viral genome quantification

KSHV genome copies were quantified as previously described (71). Genomic DNA from cells was purified with the DNeasy Blood and Tissue Kit (Qiagen, CAT#69504). An equal volume of supernatant was harvested and treated with DNase to remove free KSHV genomic DNA, and encapsidated DNA was purified to measure extracellular KSHV genome copies. The pCDNA4.TO-ORF39-2xCSTREP plasmid (72) was utilized to generate a standard curve for the cell cycle threshold (CT) versus the genome copy number. The primer sequences used to amplify the genome of KSHV were in the ORF39 region. Intracellular KSHV genomes were quantified by normalizing to the β-actin locus. Human β-actin primer forward: 5’-AAGACCTGTACGCCAACACA-3’, reverse: 5’-AGTACTTGCGCTCAGGAGGA-3’. KSHV ORF39 primer forward: 5’-GTGGGAGTATTCGTGGGTTATC-3’, reverse: 5’-GGTGAACAGTCGGAGTTCTATC-3’.

### 2.7 Western blot analyses

Cell pellets were lysed in 1X Laemmli’s protein lysis buffer and loaded in equal amounts onto SDS-polyacrylamide gels. Following semi-dry transfer (Bio-Rad), membranes were blocked in Tris-buffered saline (TBS) with 1% Tween-20 containing 5% bovine serum albumin (BSA, Fraction V) for 1 hour and then incubated overnight with primary antibodies (1:1000) at 4 °C. Membranes were washed three times with TBST for 10 minutes followed by 1 hour incubation with mouse or rabbit HRP-conjugated secondary antibody (7076/7074 Cell Signaling Technology). The following antibodies were used: anti-human β-Actin-HRP from Santa Cruz (Cat#sc-47778). The rabbit anti-Caspase 3 (#9662), anti-cleaved Caspase 3 (Asp175) (#9661), anti-cGAS (E53VW, #79978), anti-Gasdermin D (E8G3F, #97558), anti-cleaved Gasdermin D (Asp275) (E7H9G, #36425), anti-IRF3 (D6I4C, #11904), anti-phospho-IRF3 (Ser396) (4D4G, #4947), anti-MLKL (D2I6N, #14993), anti-phospho-MLKL (Ser358) (D6H3V, #91689), anti-STING (D2P2F, #13647), anti-TBK1 (E8I3G, #38066), and anti-phospho-TBK1 (Ser172) (D52C2, #5483) were purchased from Cell Signaling Technology. The anti-KSHV ORF57 antibody is from Santa Cruz Technology (cat#sc-135746 HRP). The mouse anti-KSHV interleukin-6 (vIL6) antibody was a kind gift from Dr. Blossom Damania.

### 2.8 CellTiter-Glo assay and growth curves

BCBL1, BC1, BC2, BCP1, BC3, BJAB, LCL9-23A, LCL9-23B, LCL-1, and LCL-2 cells were treated with 0.1, 1, or 10 µM diABZI for 72 hours prior to the CellTiter-Glo assay and cell counting. Live/dead cell counts were performed with trypan blue (Gibco, CAT#15250061) and a hemacytometer. BCBL1 and BC3 cells were treated with 5 µM STING inhibitor H151 (MedChemExpress cat# Hy-112693) (73) prepared in DMSO for 72 hours prior to CellTiter-Glo assay. Promega CellTiter-Glo^®^ luminescent cell viability assay was performed according to the manufacturer’s instructions (Cat#G7570) on 96-well plates.

### 2.9 Plasmid transfection

BCBL1 and BC3 cells were transfected with 50–100 ng of pcDNA3.1 or STING-HA for 48 or 72 hours using the Ingenio^®^ EZporator^®^ Electroporation System (MirusBio, cat# MIR51000) according to the manufacturer’s instructions. The STING-HA plasmid was gifted by Dr. Glen Barber (25).

### 2.10 Statistical analysis

Data analysis and graphing were performed using GraphPad Prism 5 software (GraphPad Software, San Diego, CA). All quantitative data are expressed as mean ± standard deviation. The data presented are representative of three independent experiments. A p-value less than 0.05 (p<0.05) was considered statistically significant using one-way ANOVA test or Student’s T-test.

## 3 Results

### 3.1 STING agonists activate cGAS-STING signaling in multiple KSHV-positive primary effusion lymphoma cells

**Figure 1:**
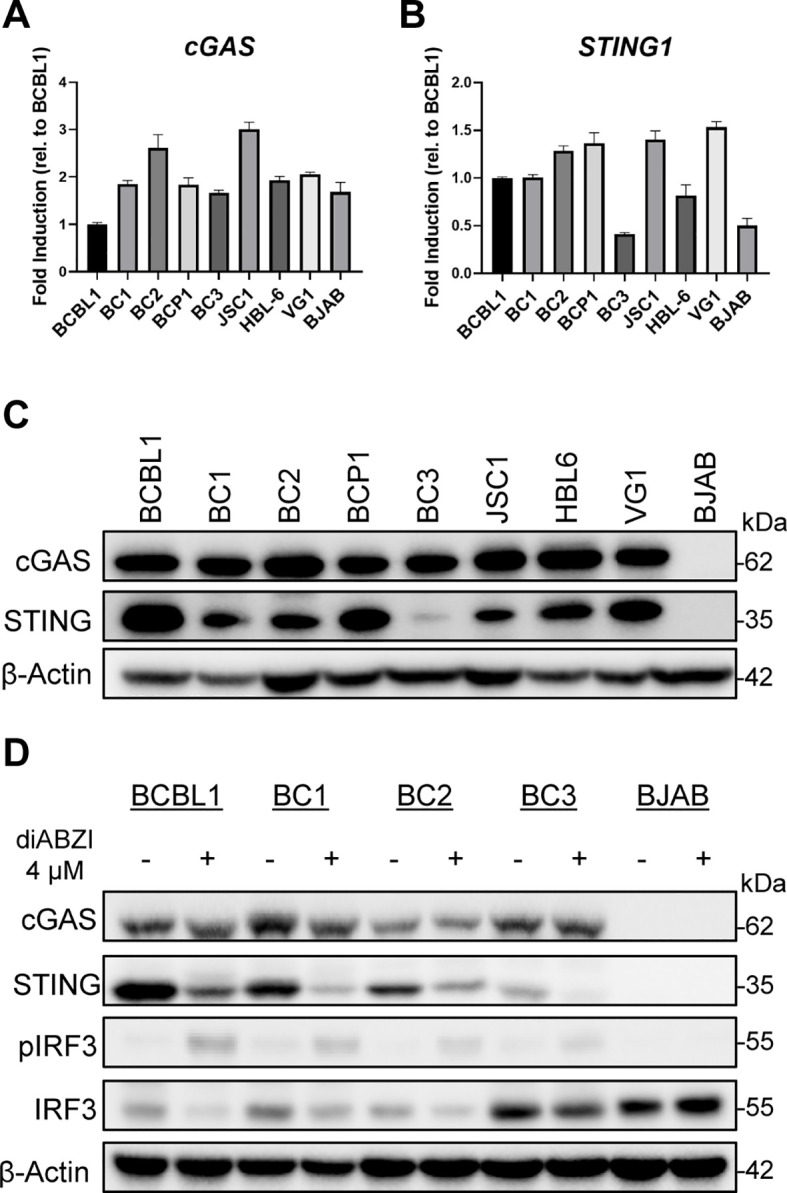
STING has variable protein expression in primary effusion lymphoma cells. RT-qPCR of **(A)** *cGAS* and **(B)** *STING1* levels across eight KSHV-positive PEL cell lines and virus-negative lymphoma BJAB cells. Fold induction for each cell line is in comparison to BCBL1. All mRNA expression levels were normalized to GAPDH. **(C)** Western blot of total cGAS and STING protein levels across PEL cell lines. **(D)** Western blot detection of cGAS and total and phosphorylated STING and IRF3 (pIRF3) in BCBL1, BC1, BC2, BC3, BJAB cells 16 hours post-treatment with 4 µM diABZI. Data **(A, B)** are presented as mean ± SD.

The cGAS-STING pathway has been shown to be suppressed in cancer cells to prevent STING-driven antitumor mechanisms (74, 75). Therefore, we first confirmed the expression of cGAS and STING in different patient-derived, KSHV-positive primary effusion lymphoma cells: BCBL1, BC1, BC2, BCP1, BC3, JSC1, HBL6, and VG1 (76–84). Across all eight PEL lines, transcript levels for *cGAS* and *STING1* were confirmed by quantitative PCR (**Figures 1A, B**). Compared to the other lines, BC2 and JSC1 had noticeably higher *cGAS* transcript levels, although protein expression was similar across all lines (**Figures 1A, C**). BC3 and the EBV-negative B-lymphoma BJAB cells had lower *STING1* expression compared to the other PEL cell lines. This pattern was reflected in the protein expression, as BC3 had minimal STING protein while BJAB lacked both cGAS or STING protein expression (**Figure 1C**). The remaining PEL cell lines expressed cGAS and STING to varying degrees, with BCBL1, BCP1, and VG1 showing the highest levels of STING protein expression. Interestingly, while *STING1* transcript levels were comparable across BCBL1, BC1, BC2, BCP1, JSC1, and VG1, protein expression was highly variable. Following confirmation of STING protein expression in the PEL cells, we investigated the ability of diABZI (49), a potent STING agonist, to activate STING signaling in PEL. BCBL1, BC1, BC2, BC3, and BJAB were directly treated with 4 µM diABZI for 16 hours before cells were harvested for RNA and protein. Activation of the STING pathway was assayed by change in STING abundance and phosphorylated IRF3 using Western blotting. STING activation was reflected by a decrease in the total STING band and the presence of a faint second upper band. We detected activated STING and phosphorylated IRF3 (pIRF3) in BCBL1, BC1, BC2, and BC3 following diABZI treatment (**Figure 1D**). As expected, there was no activation of STING or downstream signaling proteins following diABZI treatment in BJAB, which lacks STING protein.

### 3.2 STING agonists inhibit lytic replication in STING-competent BCBL1

**Figure 2:**
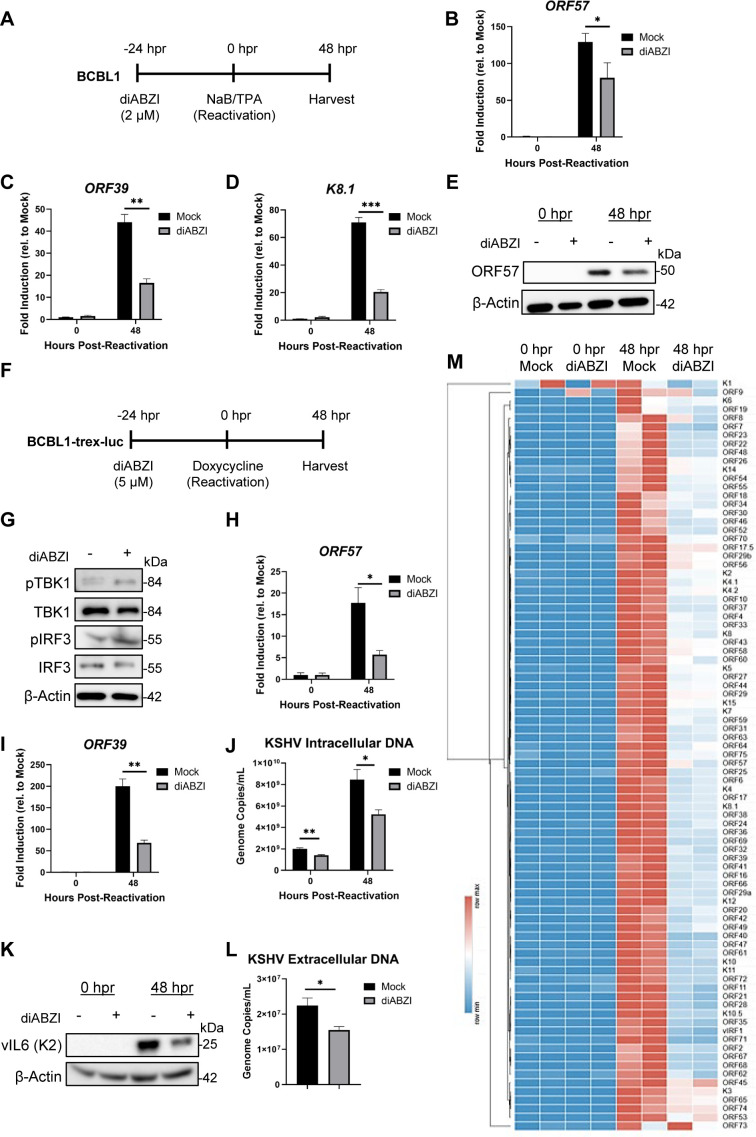
STING agonist treatment inhibits KSHV lytic replication in BCBL1 and BCBL1-TRex-luciferase cells. **(A)** Experimental schematic for data shown in **B–E**. BCBL1 cells were treated with 2 µM diABZI for 24 hours prior to reactivation with NaB (1mM) and TPA (25 ng/mL). At 48 hours post-reactivation, mRNA expression of KSHV **(B)** immediate early gene *ORF57*, **(C)** early gene *ORF39*, and **(D)** late gene K8.1 were measured by RT-qPCR. **(E)** Western blot detection of KSHV protein ORF57 at 0 and 48 hours post-reactivation. **(F)** Experimental schematic for data shown in **G–K, M**. BCBL1-TRex-luciferase cells were treated with 5 µM diABZI for 24 hours prior to reactivation with doxycycline (1ug/mL). **(G)** Western blot for cGAS-STING activation (pTBK1, pIRF3) prior to reactivation. At 48 hours post-reactivation, mRNA expression of KSHV **(H)** immediate early gene *ORF57* and **(I)** early gene *ORF39* were measured by RT-qPCR. **(J)** At 48 hours post-reactivation, genomic DNA was extracted and measured for the number of KSHV ORF39 copies/mL by qPCR, normalized to genome β-actin copies. **(K)** Western blot detection of KSHV protein vIL6 (K2) at 0 and 48 hours post-reactivation. **(L)** BCBL1-TRex-luciferase cells were pretreated with 5 µM diABZI for 6 hours prior to reactivation. Equal volumes of supernatant were harvested at 72 hours post-reactivation and extracellular virion production was measured using the KSHV ORF39 gene. **(M)** RT-qPCR-based whole KSHV transcriptome array was performed. Higher transcript expression levels are indicated by red and lower expression levels by blue as shown in the key. All transcripts normalized to 18S rRNA. Data **(B–D, H–J, L)** are presented as mean ± SD. * indicates p<0.05; ** indicates p<0.01; *** indicates p<0.001 by Student’s t-test.

Built upon the discovery that the cGAS-STING pathway restricts KSHV lytic reactivation in iSLK.219 cells (60), we are now investigating more physiological-relevant KSHV cancer models, patient-derived PEL cell lines. We pretreated BCBL1 cells with 2 µM diABZI for 24 hours prior to reactivation with sodium butyrate (NaB, 1 mM) and 12-O-tetradecanoyl phorbol-13-acetate (TPA, 25 ng/uL) as previously described (75, 84) (**Figure 2A**). At 48 hours post-reactivation, diABZI treatment reduced the transcript levels of representative KSHV immediate-early (*ORF57*), early (*ORF39*), and late (*K8.1*) genes compared with untreated controls (**Figures 2B–D**). Consistent with this, ORF57 protein expression was also reduced following agonist treatment (**Figure 2E**). While sodium butyrate and TPA are standard agents for KSHV reactivation in PEL models, both can also broadly alter host-cell transcriptional programs (85, 86). We next sought a more specific system to assess the effect of STING activation on KSHV reactivation.

We utilized a BCBL1-derived cell line, further referred to as BCBL1-trex-luc in this manuscript (66, 67). This cell line contains a doxycycline-inducible replication and transcription activator protein (RTA), the master KSHV lytic regulator protein whose induction is sufficient to reactivate KSHV. We treated BCBL1-trex-luc cells with 5 µM diABZI for 24 hours prior to reactivation by doxycycline (1 µg/mL) (**Figure 2F**). We first confirmed the activation of the STING pathway via pTBK1 and pIRF3 detection at the time point right before KSHV reactivation (**Figure 2G**). At 48 hours post-reactivation, there was a 12- and 132-fold reduction in the mRNA levels of KSHV lytic *ORF57* and *ORF39*, respectively, compared to the mock-treated cells (**Figures 2H, I**). Viral replication was decreased as indicated by a reduced KSHV genome copy number in the agonist-treated condition compared to mock (**Figure 2J**). This phenotype was further reflected by a decrease in viral KSHV protein vIL6 (K2) (**Figure 2K**). There was a reduction in virion production at 72 hours post-reactivation as quantified by extracellular KSHV encapsidated genomes (**Figure 2L**). To better evaluate the overall impact of STING activation on KSHV lytic reactivation in BCBL1, we profiled the KSHV ORF transcriptome as previously described (60, 71). Briefly, the same experiment as described in **Figure 2F** was performed and RNA was isolated at 0 and 48 hours post-reactivation then subjected to the RT-PCR-based transcriptome array targeting 88 KSHV genes. STING agonist treatment caused a global attenuation of KSHV lytic transcription compared to the mock condition (**Figure 2M**). Overall, this is consistent with our and others’ iSLK-based findings that STING is an important restriction barrier against KSHV reactivation (60, 62, 64).

### 3.3 Lytic replication in BC3 cells is minimally repressed by STING agonist treatment due to STING deficiency

**Figure 3:**
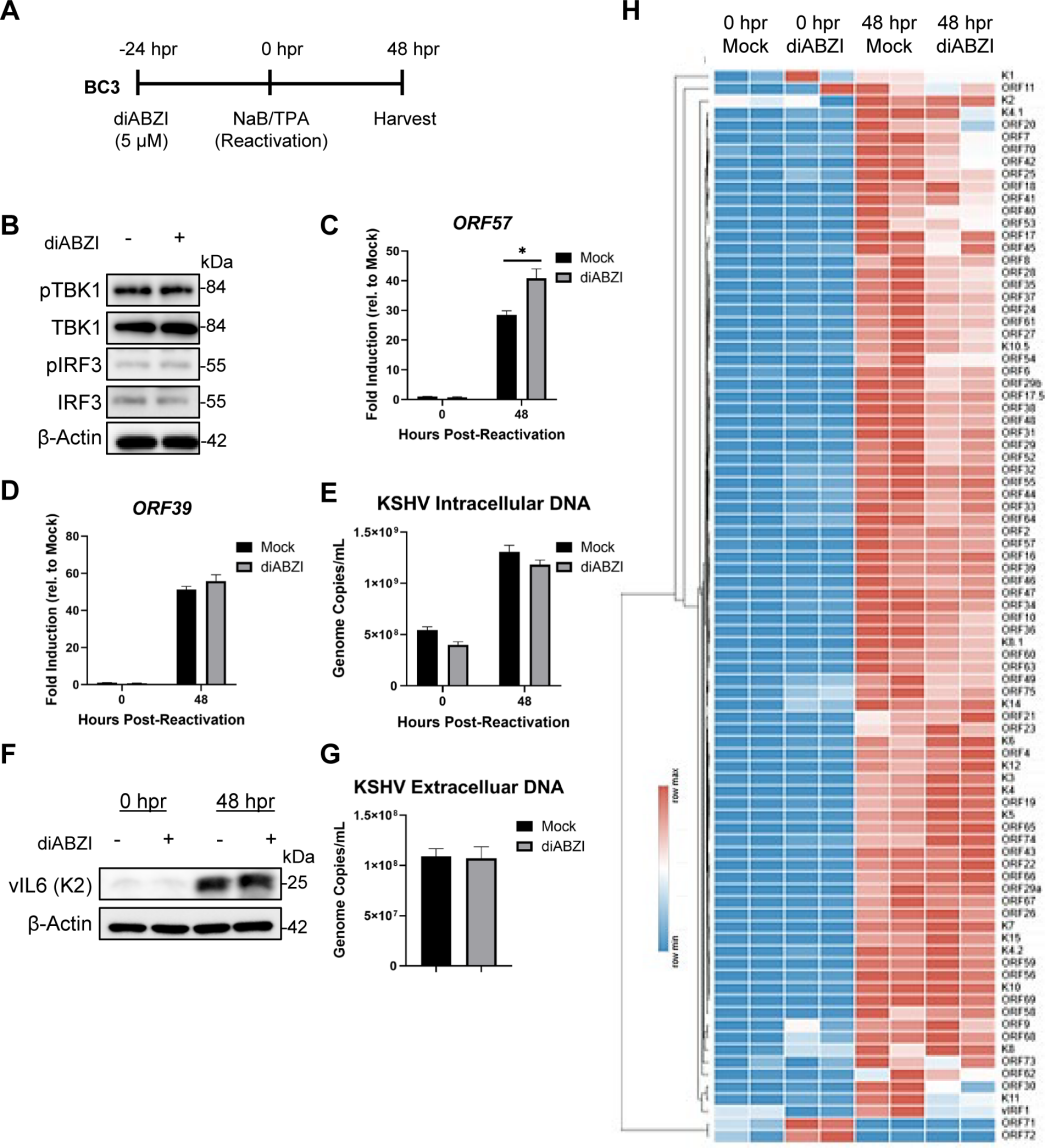
STING agonist treatment minimally inhibits KSHV lytic replication in BC3 cells. **(A)** Experimental schematic for data shown in B-F, H. BC3 cells were treated with 5 µM diABZI for 24 hours prior to reactivation with NaB (1mM) and TPA (25 ng/mL). **(B)** Western blot for cGAS-STING activation (pTBK1, pIRF3) prior to reactivation. The mRNA expression of KSHV **(C)** immediate early gene *ORF57* and **(D)** early gene *ORF39* were measured by RT-qPCR. **(E)** At 48 hours post-reactivation, genomic DNA was extracted and measured for the number of encapsidated KSHV ORF39 copies/mL by qPCR, normalized to genome β-actin copies. **(F)** Western blot detection of KSHV protein vIL6 (K2). **(G)** Equal volumes of supernatant were harvested at 48 hours post-reactivation and extracellular virion production was measured using the KSHV ORF39 gene. **(H)** RT-qPCR-based whole KSHV transcriptome array was performed. Higher transcript expression levels are indicated by red and lower expression levels by blue as shown in the key. All transcripts normalized to 18S rRNA. Data **(C-E, G)** are presented as mean ± SD. * indicates p<0.05 by Student’s t-test.

Our profiling of eight PEL cell lines revealed that BC3 has markedly reduced STING expression compared to BCBL1 (**Figures 1B, C**). In latently infected BC3 cells, treatment with diABZI was able to induce phosphorylation of STING and IRF3 (**Figure 1D**). To determine if diABZI could impact lytic reactivation in a PEL cell line with low STING expression, we performed a similar set of experiments as described in **Figure 2F**. BC3 cells were pretreated with 5 µM diABZI for 24 hours prior to reactivation with sodium butyrate (NaB, 1 mM) and 12-O-tetradecanoyl phorbol-13-acetate (TPA, 25 ng/uL) (**Figure 3A**). In contrast with BCBL1, BC3 showed only minimal induction of pTBK1 and pIRF3 upon diABZI treatment, revealing an attenuated innate immune response immediately before reactivation (**Figure 3B**). Consistently, we did not observe a significant decrease in *ORF57* or *ORF39* transcripts levels in the diABZI-treated group (**Figures 3C, D**). Rather, a slight increase in ORF57 was observed. Furthermore, STING agonist treatment had no impact on viral replication as there was no change in cellular viral genome copies was observed (**Figure 3E**). Additionally, KSHV vIL6 protein levels were unchanged (**Figure 3F**) and virion production was not impacted by agonist treatment as reflected by similar genome copy levels (**Figure 3G**). We further performed a KSHV transcriptome array and found that STING agonist treatment caused only a mild reduction on a subset of KSHV transcripts (**Figure 3H**). However, this impact was not as robust as the phenotype observed in the BCBL1-trex-luc cells, which have comparably higher STING expression. Overall, these data indicate that low baseline STING abundance in BC3 limits the ability of STING agonist treatment to mount robust downstream signaling and suppress KSHV lytic replication in this PEL model.

### 3.4 PEL cell growth and viability is inhibited by STING agonist treatment

**Figure 4:**
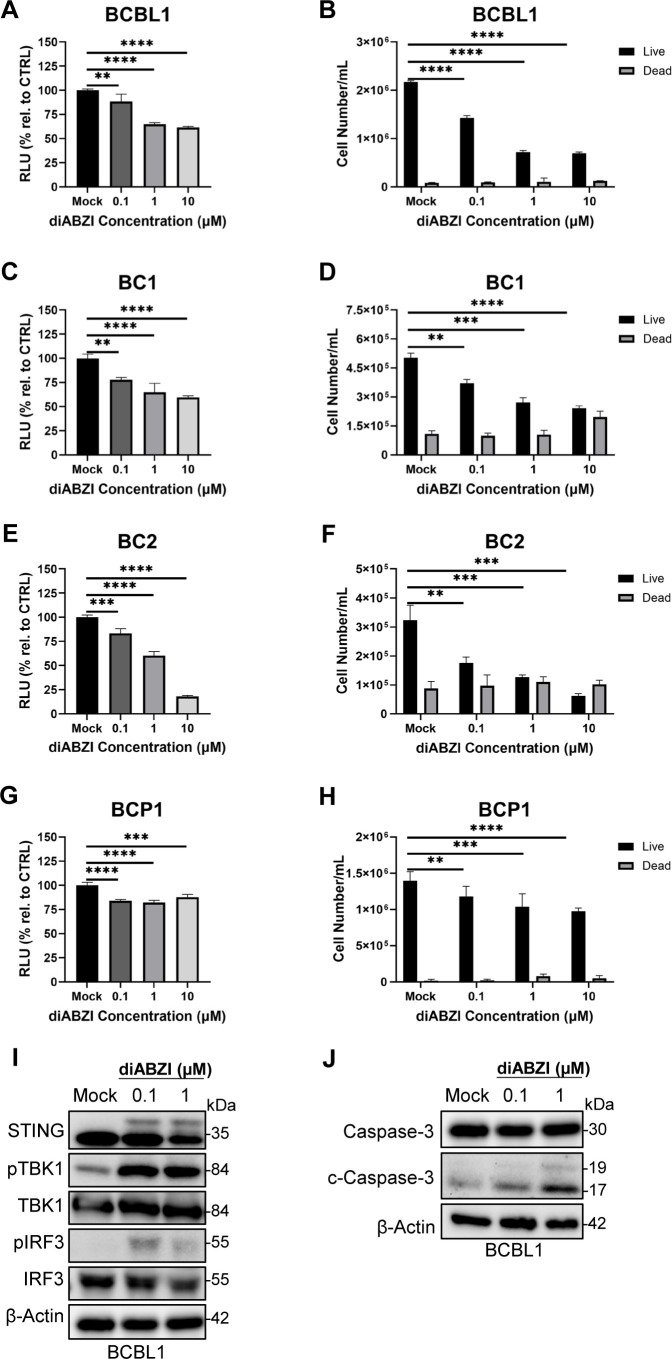
Four PEL cell lines are sensitive to STING agonist treatment. CellTiter-Glo assay in **(A)** BCBL1, **(C)** BC1, **(E)** BC2, and **(G)** BCP1 cells treated with diABZI in a dose-dependent manner for 72 hours. Data are represented as percentage of relative luminescence compared to the mock condition. Live/dead cell counts in **(B)** BCBL1, **(D)** BC1, **(F)** BC2, and **(H)** BCP1 cells treated with diABZI in a dose-dependent manner for 72 hours. **(I)** Western blot of STING-TBK1-IRF3 activation after 16 hours of diABZI treatment. **(J)** Western blot of total capase-3 and cleaved caspase-3 following 4 hours of diABZI treatment. Data **(A-H)** are presented as mean ± SD of three independent biological repeats. ** indicates p<0.01; *** indicates p<0.001; **** indicates p<0.0001 by one-way ANOVA for CellTiter-Glo and Student’s t-test for live/dead cell counts.

Because STING competence shaped the antiviral response across PEL models, we next asked whether pharmacologic STING activation similarly restricts PEL cell growth and viability during latency in a STING-dependent manner. STING agonists have been reported to slow cancer growth and induce cell death (58, 87–89). BCBL1, BC1, BC2, and BCP1 were treated with 0.1, 1, or 10 µM diABZI for 72 hours and then subjected to a CellTiter-Glo assay. This luminescence-based assay determines the amount of metabolically active cells by quantifying ATP as a measure of cell viability. After 72 hours of agonist treatment, BCBL1, BC1, BC2, and BCP1 displayed decreased cell viability in a dose-dependent manner, indicated by a reduction in relative luminescence compared to the mock condition (**Figures 4A, C, E, G**). Under the same treatment conditions, a live/dead cell count showed a reduction in cell growth in all four PEL cell lines, with BC1 and BC2 displaying the highest level of dead cell counts (**Figures 4B, D, F, H**). Notably, although BCP1 expresses relatively high STING protein (**Figure 1C**), its response to diABZI appeared more modest than the other responsive PELs in the viability and live/dead readouts (**Figures 4G, H**), suggesting that factors beyond STING abundance may influence sensitivity. We next probed for potential cell death mechanisms triggered by STING activation in PELs, such as STING-dependent apoptosis (90). Consistent with pathway engagement, diABZI induced STING-TBK1-IRF3 signaling in a dose-dependent manner in BCBL1 after 16 hours of treatment (**Figure 4I**). Indeed, after 4 hours of diABZI treatment, we found increased levels of cleaved caspase-3 in BCBL1 cells, suggesting that apoptosis contributes to diABZI-mediated cytotoxicity responsive PELs (**Figure 4J**).

### 3.5 BC3 is resistant to STING agonist treatment

**Figure 5:**
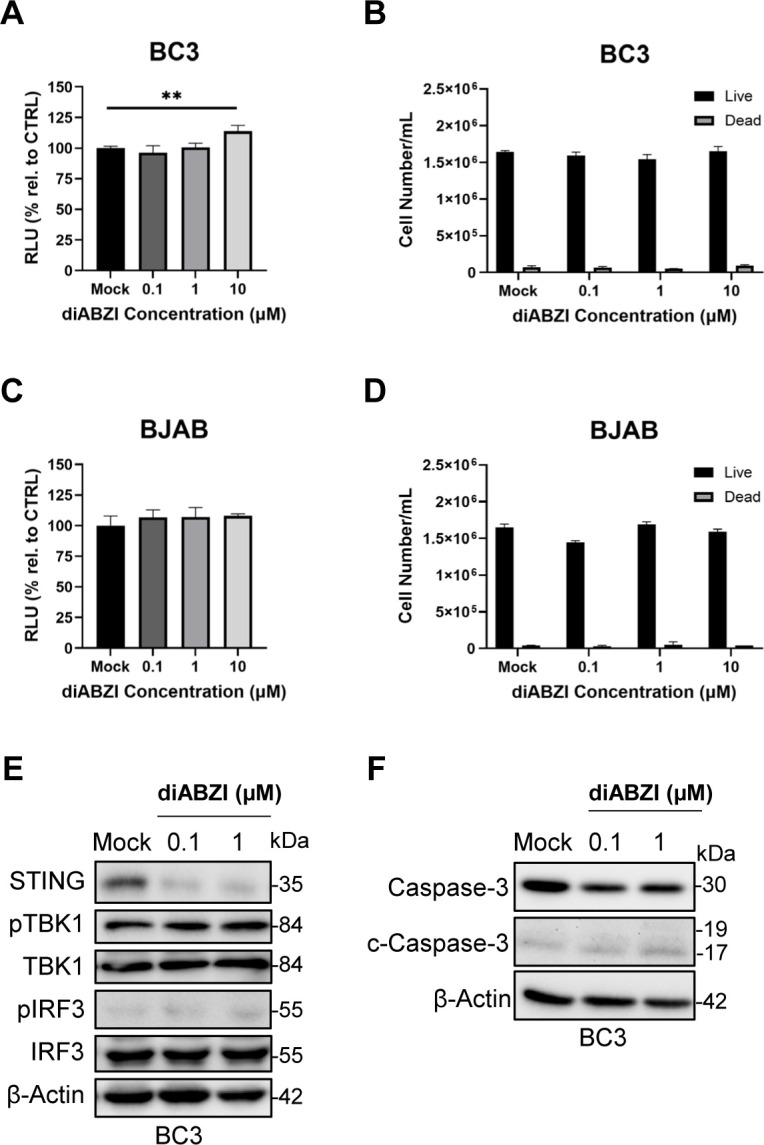
BC3 is resistant to STING agonist treatment. **(A)** BC3 and **(C)** BJAB cells treated with diABZI in a dose-dependent manner for 72 hours were subjected to CellTiter-Glo assay. Data is presented as percentage of relative luminescence compared to the mock condition. Live/dead cell counts in **(B)** BC3 and **(D)** BJAB cells treated with diABZI in a dose-dependent manner for 72 hours. **(E)** Western blot of STING-TBK1-IRF3 activation after 16 hours of diABZI treatment. **(F)** Western blot of total caspase-3 and cleaved caspase-3 following 4 hours of diABZI treatment. Data **(A-D)** are presented as mean ± SD of three independent biological repeats. ** indicates p<0.01 by one-way ANOVA for CellTiter-Glo and Student’s t-test for live/dead cell counts.

To assess whether this growth restriction requires STING pathway competence, we next evaluated STING^low^ or STING-deficient B-cell lymphoma lines under the same diABZI treatment conditions. We performed identical assays on the low STING-expressing BC3 and STING-deficient BJAB and observed no decrease in cell viability (**Figures 5A, C**) or cell growth (**Figures 5B, D**). In BC3, a STING activation signature was detectable by Western blot (i.e., changes in total STING), but downstream TBK1-IRF3 activation and cleaved caspase-3 were not observed (**Figures 5E, F**). Based on these results, diABZI treatment can reduce PEL cell viability and cell growth but is limited by compromised STING expression and its downstream signaling.

Since STING is implicated in multiple cell death pathways (41, 89, 91–93), we sought to evaluate additional mechanisms involved in our anti-proliferative phenotype. In BCBL1 cells, diABZI treatment was associated with a slight increase in cleaved Gasdermin D, whereas this pattern was not observed in BC3 cells (**Supplementary Figure 1**), suggesting that pyroptotic signaling may be modestly engaged under these conditions. Incontrast, phosphorylated MLKL was not induced in either BCBL1 or BC3 following agonist treatment(**Supplementary Figure 2**), arguing against detectable activation of necroptosis in this setting. Together, these findings suggest that diABZI-mediated growth inhibition in PEL cells may involve multiple downstream mechanisms, while the relative contribution of each pathway remains to be defined.

### 3.6 STING loss- and gain-of-function studies support a growth-restraining role in PEL

**Figure 6:**
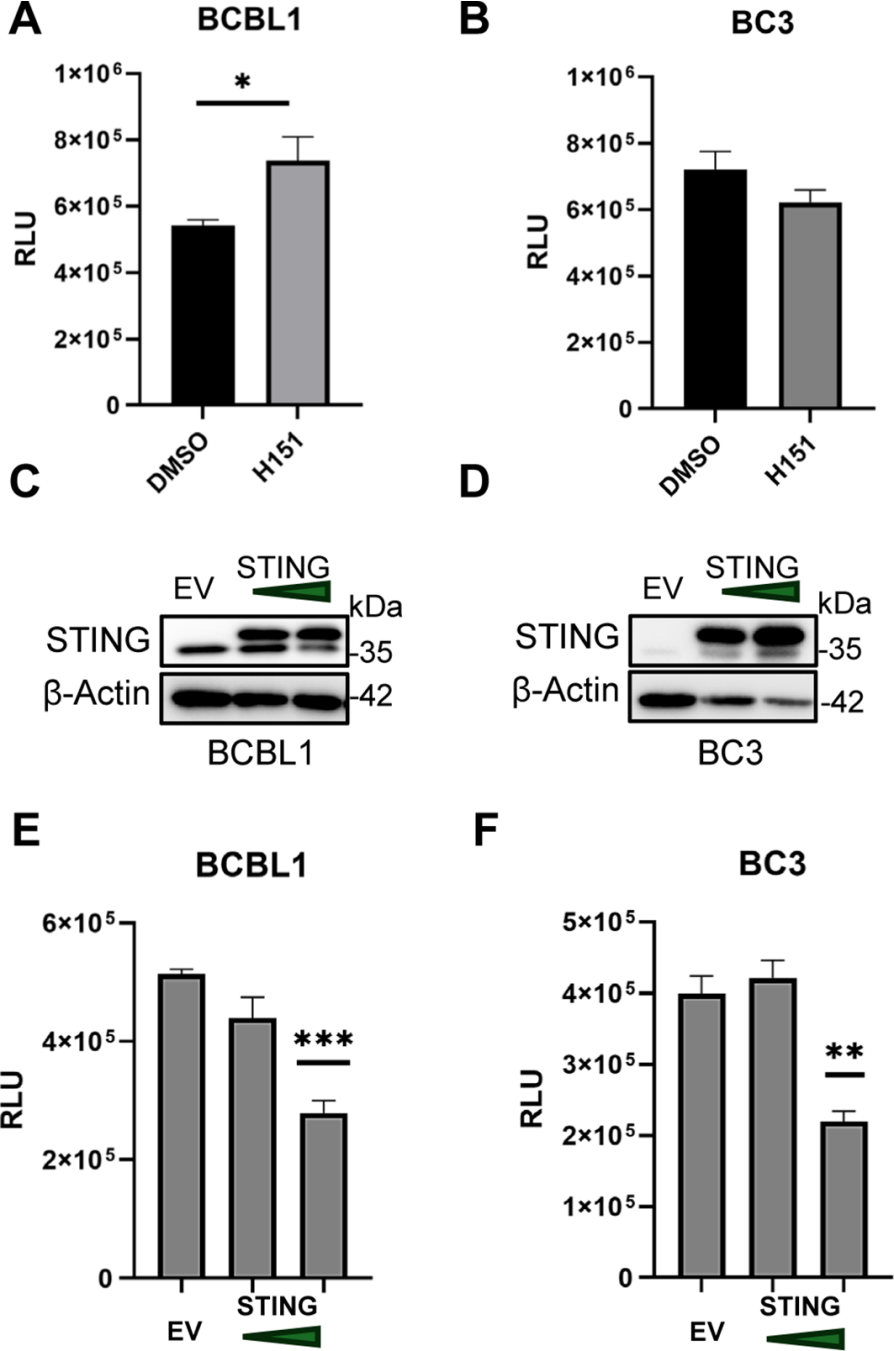
STING loss- and gain- of function studies support a growth-restraining role in PEL. **(A)** BCBL1 and **(B)** BC3 cells were treated with 5 µM H151 for 72 hours and subjected to CellTiter-Glo assay. Western blot of STING overexpression 48 hours after electroporation of 100 ng pcDNA3.1 (EV) or 50-100 ng STING plasmid in **(C)** BCBL1 and **(D)** BC3 cells. CellTiter-Glo assay in **(E)** BCBL1 and **(F)** BC3 cells at 72 hours after EV or STING plasmid electroporation. Data is represented as relative luminescence units. Data **(A-B, E-F)** are presented as mean ± SD. * indicates p<0.05; ** indicates p<0.01; *** indicates p<0.001 by Student’s t-test.

To further address whether STING directly regulates PEL cell growth, we performed complementary loss- and gain-of-function experiments in BCBL1 and BC3 cells. In BCBL1 cells, pharmacologic inhibition of STING with H151 (73) significantly increased cell growth after 72 hours, consistent with the idea that basal STING activity restrains proliferation under baseline conditions (**Figure 6A**). In contrast, H151 did not provide a growth advantage in BC3 cells, supporting the interpretation that endogenous STING activity is already markedly limited in this STING^low^ model (**Figure 6B**).

To determine whether increasing STING expression is sufficient to suppress PEL growth, we ectopically expressed STING in BCBL1 and BC3 cells. Western blot analysis confirmed successful STING overexpression in both cell lines (**Figures 6C, D**). In BCBL1 cells, STING overexpression reduced cell growth in a dose-dependent manner (**Figure 6E**). Notably, restoration of STING in BC3 cells also caused marked growth inhibition, with the highest STING dose producing a pronounced reduction in luminescence (**Figure 6F**). Together, these reciprocal perturbation experiments support a tumor cell-intrinsic, growth-restraining role for STING in PEL and further indicate that the STING^low^ state in BC3 is functionally linked to resistance to STING-mediated cytotoxicity.

### 3.7 Activation of STING signaling in EBV-positive lymphoblastoid cell lines inhibits cell growth and viability

**Figure 7:**
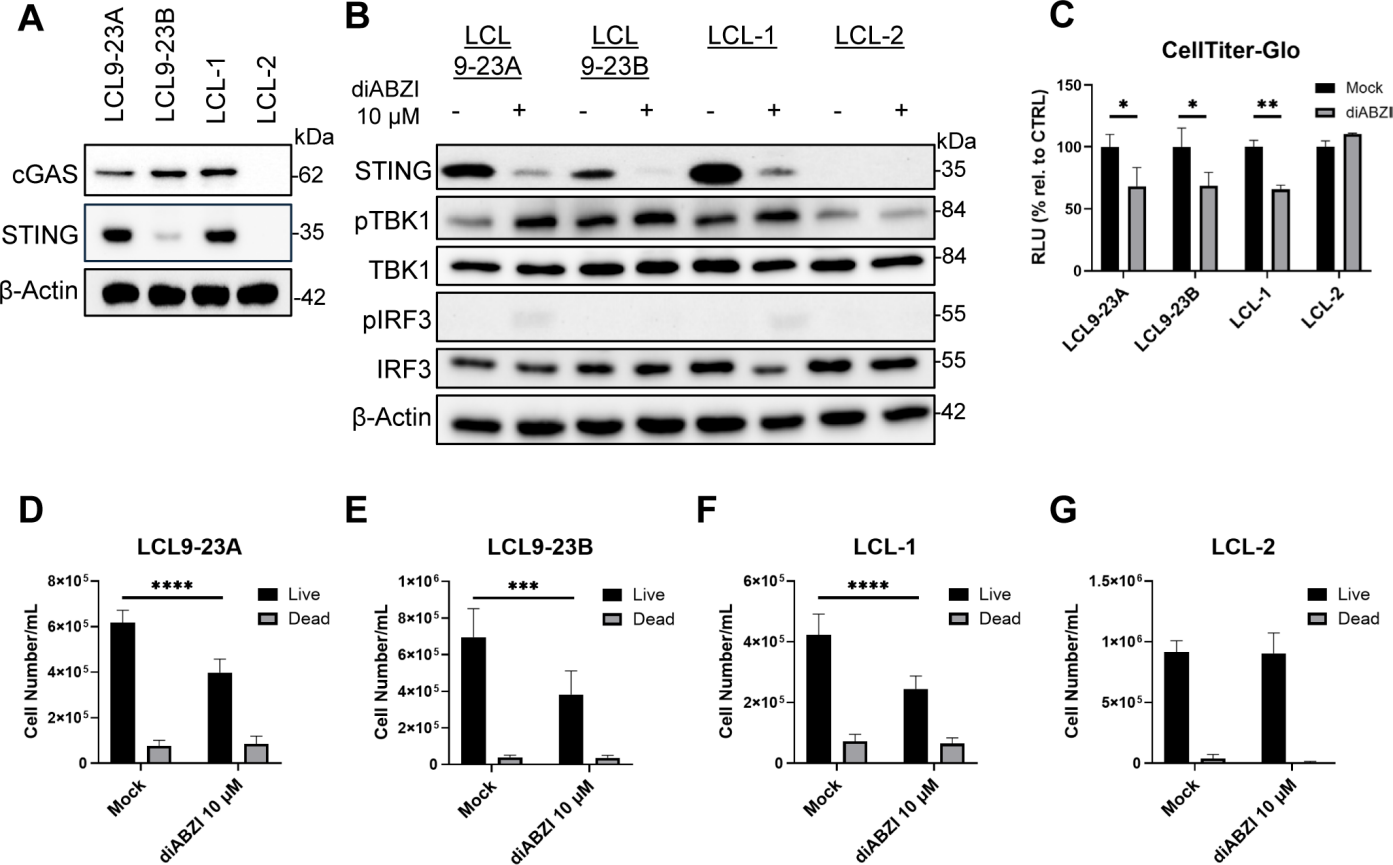
STING activation and cell growth inhibition by diABZI in four EBV-transformed lymphoblastoid cell lines. **(A)** Western blot of four LCLs for total cGAS and STING. **(B)** Western blot of LCLs after 5 hours of 10 µM diABZI treatment. Activation of STING is reflected by the degradation of total STING, increase in phosphorylated TBK1 (pTBK1), and presence of phosphorylated IRF3 (pIRF3). **(C)** CellTiter-Glo assay of four LCLs after 72 hours of 10 µM diABZI treatment. Data represented as percent of relative luminescence compared to mock (control). Live/dead cell counts of **(D)** LCL9-23A, **(E)** LCL9-23B, **(F)** LCL-1, and **(G)** LCL-2 following 72 hours of 10 µM diABZI treatment. Data **(C-G)** are presented as mean ± SD. * indicates p<0.05; ** indicates p<0.01; *** indicates p<0.001; **** indicates p<0.0001 by Student’s t-test.

Since diABZI-mediated cytotoxicity tracked with STING pathway competence in PEL, we asked whether this relationship generalizes to other gammaherpesvirus-associated B-cell models. A related gammaherpesvirus that causes infectious mononucleosis, Epstein-Barr virus (EBV), infects about 95% of global adults (94, 95). EBV causes a wide variety of malignant diseases, including Burkitt lymphoma, Hodgkin lymphoma, post-transplant lymphoproliferative diseases (PTLD), diffuse large B cell lymphoma (DLBCL), and nasopharyngeal cell carcinoma. EBV utilizes multiple strategies to evade host innate immune signaling pathways, including the cGAS-STING-TBK1 axis (96). Based on the results of diABZI treatment in the PEL cell lines, we aimed to investigate the impact of STING activation in EBV-transformed lymphoblastoid cell lines (LCLs) (68–70), which are an important model for PTLD and DLBCL. First, we confirmed the presence of cGAS and STING in four different LCLs, LCL9-23A, LCL9-23B, LCL-1, and LCL-2. LCL9-23A and LCL-1 had similar levels of STING protein while LCL9-23B was highly reduced (**Figure 7A**). To test pathway functionality, we treated all four LCLs with 10 µM diABZI for 5 hours and assessed STING pathway activation. STING activation (reflected by degradation/shift of total STING) and downstream signaling (increased pTBK1 and pIRF3) were detected in LCL9-23A, LCL9-23B, and LCL-1 but not in LCL-2 (**Figure 7B**). As expected, LCL-2 did not induce pSTING, pTBK1, or pIRF3 following STING agonist treatment due to the lack of STING protein. After confirming STING pathway competence, we sought to evaluate the impact of a STING agonist on the cell growth and cell viability in LCLs. All four cell lines were treated with 10 µM diABZI for 72 hours and subjected to both a CellTiter-Glo assay and live/dead cell count (**Figures 7C–G**). The agonist-treated samples in LCL9-23A, LCL9-23B, and LCL-1 showed decreased viability while LCL-2 cell viability was not impacted (**Figure 7C**). After 72 hours, LCL9-23A, LCL9-23B, and LCL-1 had a significant decrease in cell growth (**Figures 7D–F**). The cell growth of LCL-2 was not reduced by diABZI due to the lack of STING, mirroring the cell viability phenotype (**Figure 7G**). Collectively, our data suggest STING agonist treatment can activate the STING pathway and inhibit cell growth in EBV-transformed LCLs, with responsiveness aligning with STING pathway competence.

## 4 Discussion

The cGAS-STING pathway is critical for antiviral defense and tumor control, linking cytosolic DNA sensing with downstream TBK1-IRF3 signaling programs that can restrict pathogens and constrain malignant cell growth (24, 25, 37, 74, 97). In parallel, pharmacologic STING agonists have emerged as a rapidly developing area in cancer immunotherapy and antiviral research, with multiple agents in clinical trials for non-viral cancers (44, 45). However, how tumor cell intrinsic STING pathway competence shapes both viral replication programs and lymphoma cell growth in gammaherpesvirus-associated malignancies remains incompletely defined. Kaposi’s sarcoma-associated herpesvirus (KSHV) establishes lifelong infection and drives malignancies in which both latent oncogenic programs and lytic reactivation can contribute to pathogenesis, yet there are not clinically approved KSHV-specific vaccines or curative therapies (8, 9). Here, we used diABZI to assess STING pathway competence in patient-derived PEL models. STING activation was associated with suppression of KSHV lytic reactivation and reduced lymphoma cell growth in STING-responsive cell lines, whereas models with minimal STING protein expression exhibited minimal responses under the same conditions. Extending this concept to EBV-transformed lymphoblastoid cell lines, responsiveness to diABZI similarly aligned with preservation of the cGAS-STING pathway, supporting the idea that STING functions as a host-intrinsic barrier with both antiviral and antitumor impacts in KSHV-associated B-cell models.

Because STING signaling can impose both antiviral and antitumor constraints, multiple cancers attenuate this pathway through genetic alterations and/or epigenetic silencing (25, 74, 75, 98–100). Consistent with this concept, we observed substantial heterogeneity in cGAS-STING pathway status across eight patient-derived PEL cell lines. While *STING1* transcripts were detectable across the panel, STING protein abundance varied markedly, with BCBL1, BCP1, and VG1 exhibiting the highest STING protein expression, and BC3 showing reduced *STING1* transcripts and minimal STING protein. This heterogeneity could reflect epigenetic attenuation mechanisms reported across multiple tumor types, although this will require direct validation in PEL models. Notably, except for BC3, *STING1* mRNA levels were broadly comparable among several PEL lines (BCBL1, BC1, BC2, BCP1, JSC1, and VG1) despite highly variable STING protein. Several mechanisms might contribute to this discordance between *STING1* transcript and STING protein, including post-transcriptional and post-translational regulation. For example, KSHV can directly repress STING post-transcriptionally via viral miRNAs (miR-K12-6-3p, miR-K12-7-3p, and miR-K12-11-3p) (65). Cellular miRNAs have been reported to target and repress STING in other settings, including miR-576-3p, miR-24, and miR-181a (101–103). Steady-state STING protein abundance and signaling output can also be shaped by activation-coupled trafficking/modification requirements (e.g., ER-to-Golgi trafficking and Golgi-associated palmitoylation required for productive downstream signaling) (104, 105). The basis for the STING^low^ phenotype in BC3 remains unresolved in the current study and is likely multifactorial. Because BC3 is a patient-derived PEL line, pre-existing tumor heterogeneity cannot be excluded. In addition, KSHV encodes miRNAs that directly repress STING1 translation, and BC3 has been reported to contain an unusually high proportion of viral miRNAs, making viral post-transcriptional suppression a plausible contributor (65, 106). Epigenetic silencing is another attractive possibility, as STING repression by promoter methylation has been documented across multiple cancers (98, 99). Future studies examining *STING1* methylation, genomic status, and viral miRNA contribution in BC3 will be needed to distinguish among these non-mutually exclusive mechanisms. Together, these observations support the idea that tumor cell intrinsic STING is heterogeneous in PEL and might influence functional responses to pharmacologic STING activation.

We next evaluated whether STING activation could function as an intrinsic barrier to KSHV lytic reactivation. In BCBL1 cells, diABZI pretreatment suppressed lytic reactivation, while exhibiting minimal antiviral effects in STING^low^ BC3, consistent with limited downstream TBK1-IRF3 pathway output. Given that human B cells have been reported to secrete little to no type I interferon after cytosolic DNA stimulation, we prioritized proximal signaling readouts (TBK1-IRF3 phosphorylation) over secreted interferon endpoints (107). Together, these data suggest that suppression of KSHV lytic reactivation by STING activation depends on tumor cell-intrinsic STING pathway competence and is not uniformly observed across PEL cells.

Beyond restricting KSHV lytic reactivation, our data was consistent with a cell-intrinsic growth-inhibitory effect of STING activation in PEL. Treatment with diABZI reduced growth and metabolic viability in multiple PEL cell lines (BCBL1, BC1, BC2, and BCP1), whereas STING^low^ or STING-deficient models (BC3 and BJAB) were comparatively resistant, consistent with the idea that therapeutic response requires sufficient STING pathway competence in the tumor cell. Mechanistically, STING activation has been linked to multiple cell death programs, and the induction of cleaved caspase-3 in BCBL1 following diABZI treatment is consistent with engagement of an apoptotic response in at least a subset of STING-competent PEL contexts (90, 91). In addition to cleaved caspase-3 in BCBL1, we also detected a modest increase in cleaved Gasdermin D, whereas phosphorylated MLKL was not induced under the same conditions. These data indicate that multiple growth-inhibitory or cell death-associated pathways may be engaged following STING activation, although their relative contributions are not yet fully resolved. Given that STING signaling can also promote autophagy and senescence (40, 42, 43, 108–110), additional work is needed to define the dominant downstream effector mechanisms and determine whether distinct cell death or growth-arrest programs account for the variable sensitivity observed across PEL models.

Importantly, in BCBL1 cells, pharmacologic STING inhibition with H151 increased growth, whereas ectopic STING expression reduced growth in a dose-dependent manner. Restoration of STING in BC3 also caused marked cytotoxicity, supporting the idea that reduced STING expression in this model is functionally linked to escape from intrinsic growth-inhibitory signaling. Direct STING perturbation experiments during lytic reactivation were technically challenging under our current conditions, likely due to the combined stress of transfection, diABZI treatment, and KSHV reactivation. Clarifying the direct contribution of STING to KSHV lytic reactivation will be an important goal of future studies using optimized perturbation conditions.

Notably, STING protein abundance was not fully predictive of phenotypic output, as BCP1 expressed high STING protein levels yet showed a more modest growth and viability reduction than the most sensitive STING-expressing lines in our panel. This highlights that in KSHV-associated malignancies, cGAS-STING pathway output may be further tuned by viral immune evasion mechanisms, including factors that block STING signaling downstream of detectable STING protein. For example, KSHV vIRF1 and ORF48 can antagonize STING signaling by binding STING and supporting efficient lytic replication (60, 62). KSHV also encodes additional redundant inhibitors targeting the broader DNA-sensing axis (e.g., ORF52 or cLANA inhibition of cGAS enzymatic activity), highlighting a multi-node strategy to block pathway signaling (63, 64). In parallel, host cells also encode multiple layers of negative regulation that constrain STING signaling, providing further opportunities for tumor- or virus-driven rewiring to yield STING-positive but functionally blunted phenotypes (111–113). Together, these additional factors may be considered and explored in future studies.

To assess the generalizability of STING activation beyond KSHV-driven PEL, we examined EBV-transformed lymphoblastoid cell lines and found heterogeneous cGAS-STING pathway status, including reduced STING in LCL9-23B and loss of detectable cGAS-STING in LCL-2. As expected, STING-expressing LCLs showed reduced growth and viability to diABZI treatment. In contrast, STING-deficient LCL-2 was unresponsive, paralleling to the resistance phenotype observed in STING-deficient BJAB cells. These results support STING pathway competence as one factor associated with varied responses to STING activation across virus-associated B-cell models. Because EBV has been reported to suppress cGAS-STING signaling (96), defining how EBV immune evasion and/or host-intrinsic silencing shapes STING competence will be important for understanding if STING agonists might be effective in EBV-driven disease.

Although our data establishes an association between STING pathway competence, suppression of KSHV lytic reactivation and PEL cell growth inhibition, the precise downstream effector mechanism(s) remains fully resolved. STING signaling is known to activate multiple antiviral/antitumor programs, including canonical type I interferon and ISG induction, as well as cell-intrinsic pathways such as apoptosis and autophagy (24, 43, 90, 91, 108, 114). Our findings do not distinguish whether the observed antiviral/antitumor effects primarily reflect interferon-dependent ISG expression, other direct antiviral transcriptional programs, or cell-intrinsic stress responses (115). Future studies will be needed to dissect the relative contributions of these pathways, including secreted IFN measurements, ISG profiling, and temporal analysis of cell death signaling during reactivation.

Our findings also highlight several directions for further clarifying STING biology in KSHV-associated malignancies. The variable responsiveness to diABZI observed across PEL lines supports the idea that patient-derived heterogeneity can be informative, and it suggests that STING pathway competence may contribute to differences in phenotypic output across models. The minimal response of BC3 and BJAB, which exhibit low/absent STING expression, is consistent with this interpretation and helps frame pathway attenuation as one plausible determinant of experimental outcome. In parallel, because STING activation can be accompanied by strong pro-inflammatory cytokine programs, extending these studies into settings that better capture inflammatory biology could be valuable. This is especially relevant given that Kaposi’s sarcoma can be associated with high viremia and elevated cytokines (IL-6, IL-10, and IL-1β) and that KSHV inflammatory cytokine syndrome (KICS) represents an extreme inflammatory state linked to lytic-phase cytokine release (116–119). Incorporating these variables may help contextualize STING-associated phenotypes within the broader immunopathology of KSHV disease.

Together, these data establish an *in vitro* framework linking cell-intrinsic cGAS-STING pathway competence with KSHV lytic reactivation phenotypes and lymphoma cell fitness across KSHV B-cell models. Importantly, these findings are based primarily on *in vitro* cell line models, and future studies in more physiological systems will be needed to determine whether STING agonism produces sustained antitumor activity *in vivo* and how such effects balance against potential pro-inflammatory or toxic responses.

## Data Availability

The raw data supporting the conclusions of this article will be made available by the authors, without undue reservation.

## Supplementary material

Supplementary Figure 1 STING agonist treatment triggers pyroptosis in BCBL1 cells. **(A)** BCBL1 and **(B)** BC3 cells were treated with diABZI in a dose-dependent manner for 16 hours. Western blot for total Gasdermin D (GSDMD) and cleaved Gasdermin D (c-GSDMD).

Supplementary Figure 2 STING agonist treatment does not induce necroptosis in PEL cells. **(A)** BCBL1 and **(B)** BC3 cells were treated with diABZI in a dose-dependent manner for 16 hours. Western blot for total MLKL and phosphorylated MLKL.
