# Supplementary figures and images for: STING inhibits viral lytic reactivation and cell growth in primary effusion lymphoma
Source: Front Immunol. 2026 May 5;17:1823240. doi: 10.3389/fimmu.2026.1823240 (PMC13183639; doi:10.3389/fimmu.2026.1823240)

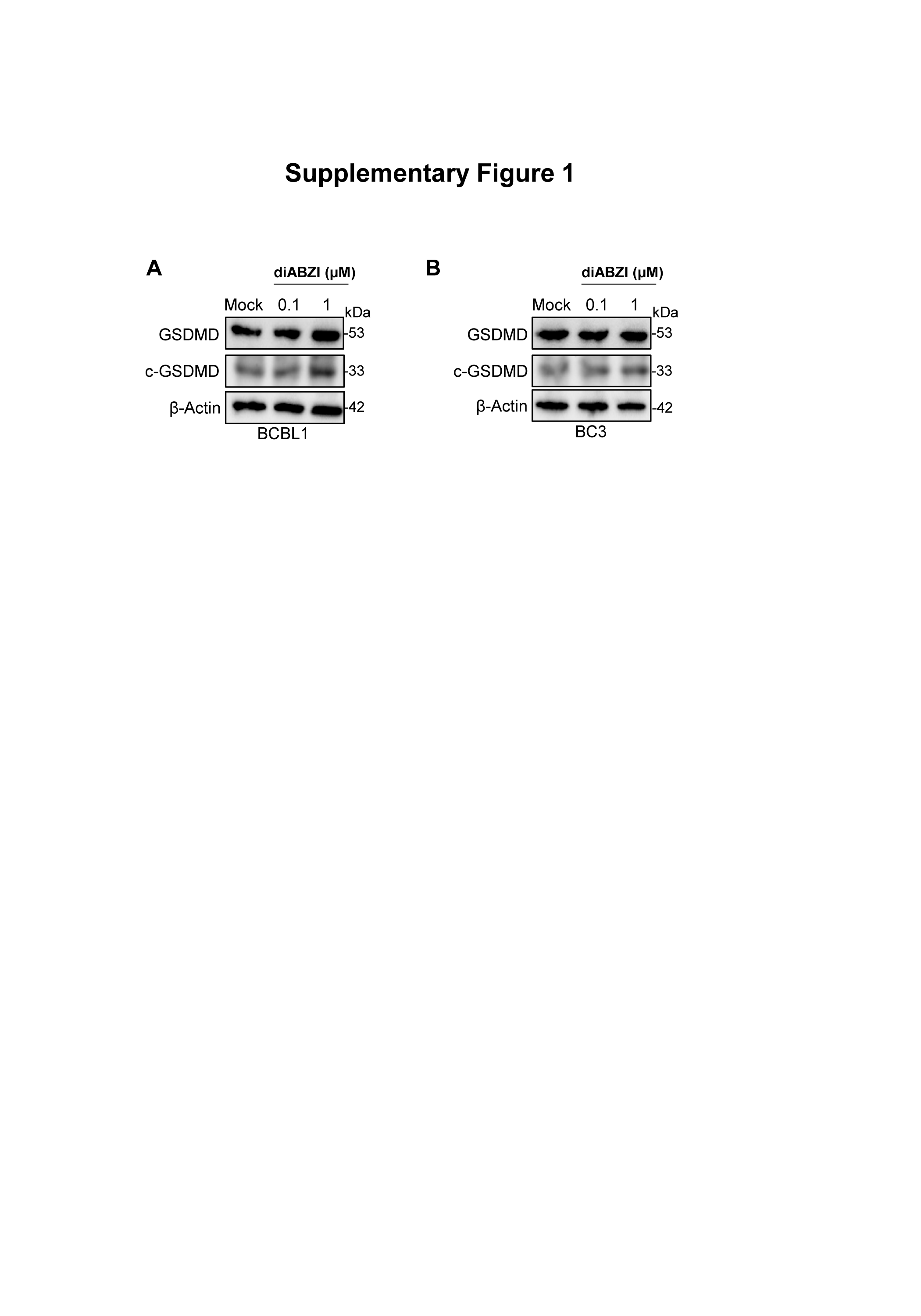

Supplement: Supplementary Figure 1 — STING agonist treatment triggers pyroptosis in BCBL1 cells. (A) BCBL1 and (B) BC3 cells were treated with diABZI in a dose-dependent manner for 16 hours. Western blot for total Gasdermin D (GSDMD) and cleaved Gasdermin D (c-GSDMD). [file Image1.tif]

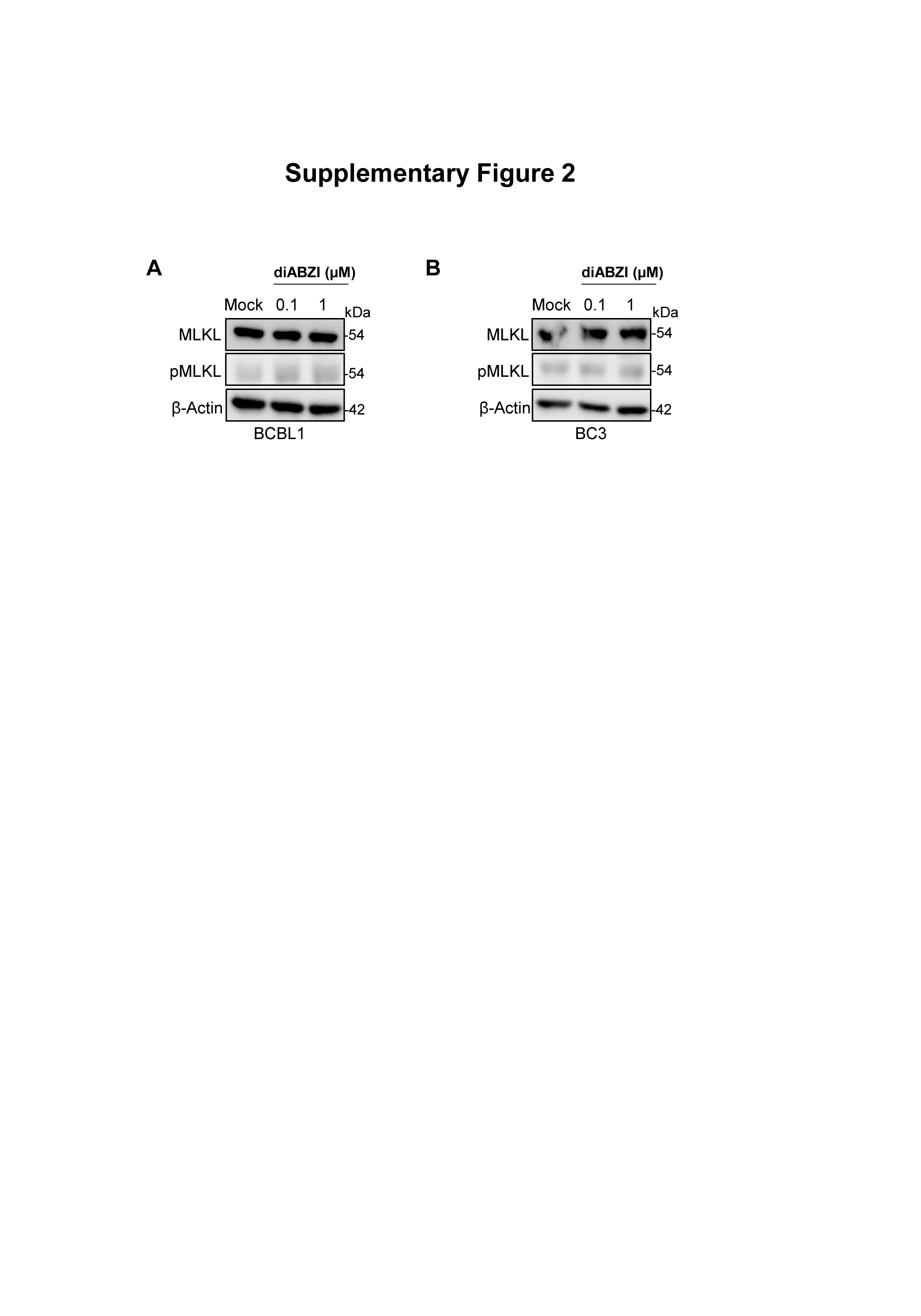

Supplement: Supplementary Figure 2 — STING agonist treatment does not induce necroptosis in PEL cells. (A) BCBL1 and (B) BC3 cells were treated with diABZI in a dose-dependent manner for 16 hours. Western blot for total MLKL and phosphorylated MLKL. [file Image2.tif]
